# *QuickStats:* Death Rates* Attributed to Excessive Cold or Hypothermia^†^ Among Persons Aged ≥15 Years, by Urbanization Level^§^ and Age Group — National Vital Statistics System, 2015–2017

**DOI:** 10.15585/mmwr.mm6807a8

**Published:** 2019-02-22

**Figure:**
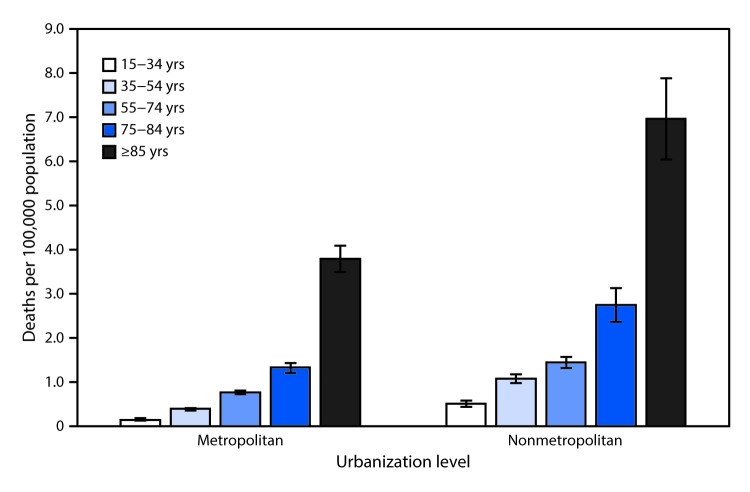
During 2015–2017, death rates attributed to excessive cold or hypothermia increased steadily with age among those aged ≥15 years in both metropolitan and nonmetropolitan counties. The rate for persons aged ≥85 years reached 3.8 deaths per 100,000 in metropolitan counties and 7.3 in nonmetropolitan counties. The lowest rates were among those aged 15–24 years (0.2 in metropolitan counties and 0.5 in nonmetropolitan counties). In each age category, death rates were lower in metropolitan counties and higher in nonmetropolitan counties.

**Source:** National Center for Health Statistics, National Vital Statistics System, Mortality Data 2015–2017.

